# Bio-Inspired Internal Representations of Tactile Sensation, Pain, and Damage for Artificial Skin Using Spatio-Temporal Anomaly Detection

**DOI:** 10.3390/s26103125

**Published:** 2026-05-15

**Authors:** Shinnosuke Fukagawa, Mitsuharu Matsumoto

**Affiliations:** 1 Department of Informatics, The University of Electro-Communications, 1-5-1, Chofugaoka, Chofu-shi 182-8585, Tokyo, Japan; f2630124@gl.cc.uec.ac.jp; 2 Graduate School of Informatics and Engineering, University of Electro-Communications, 1-5-1, Chofugaoka, Chofu-shi 182-8585, Tokyo, Japan

**Keywords:** artificial skin, nociception, damage detection, anomaly detection, spatio-temporal features

## Abstract

In recent years, the deployment of robots in human-centric environments has necessitated the development of artificial skins that integrate safety and durability. Traditional damage detection often relies on raw signal thresholds, lacking the functional integration of touch, pain, and damage found in biological systems. This study proposes a bio-inspired artificial skin model that separately evaluates these three states through a spatio-temporal anomaly detection framework. We developed an unsupervised model combining a Convolutional Autoencoder (CAE) and Convolutional LSTM (ConvLSTM) to learn the latent representations of tactile maps from intact skin. By quantifying spatial reconstruction and temporal prediction errors, the system generates individual scores for touch, pain, and damage. Pain is defined as an abstract signal of instantaneous abnormality, while damage is identified as a persistent structural deviation. We implemented a dynamic thresholding mechanism mimicking biological sensitization and recovery, with damage detection gated by a pain-flag constraint to minimize false positives. Experimental results across various conditions—including incisions (3–6 cm) and abrasions (10–30 times)—demonstrate that the model can distinguish between momentary noxious stimuli and sustained structural degradation. Quantitative evaluation shows that the proposed model achieves an Area Under the Curve (AUC) of 0.653, outperforming a threshold-based baseline and maintaining zero false positives under strong, non-damaging contact. Specifically, the system successfully mimics biological aftereffects and the pain-gating mechanism, where damage is only assessed in the presence of a pain-related trigger. This research provides a scalable, software-driven foundation for robot self-protection that overcomes the implementation constraints of hardware-dependent neuromorphic systems.

## 1. Introduction

In recent years, interest in robots that coexist with humans, including humanoid robots, has grown rapidly. These robots are expected to be used not only in industrial environments but also in situations where they directly collaborate with humans, such as in medical care, nursing care, and everyday life. For robots designed for such human interaction, it is more important than ever to use artificial skin with similar functions to human skin. Human skin has the sense of touch, which detects light touch and vibration, and the sense of pain, which detects harmful stimuli from the outside world and prevents physical damage [1]. Robots that coexist with humans are also required to have mechanisms similar to these senses of touch and pain.

Numerous tactile sensors have been developed. These sensors are designed based on various principles with the aim of accurately measuring contact position, force magnitude, slippage, and deformation that occur when a robot interacts with the outside world. Examples include flexible tactile sensors that can detect pressure and contact position [2] and optical tactile sensors that can capture material deformation with high spatial resolution [3]. In particular, capacitive tactile sensors are being developed for robot skin applications due to their ability to capture minute pressure changes [4] and temporal transitions of contact at multiple points [5], as well as their relatively simple structure and ease of expansion and expansion to larger areas [6,7]. However, while these sensors excel at raw signal acquisition, they primarily function as passive transducers. A critical limitation remains in their data processing paradigms: most existing systems lack the sophisticated logic required to internally distinguish between innocuous touch, imminent danger (pain), and actual structural damage. This deficiency often forces robot systems to rely on simplistic signal thresholding, which fails to capture the multi-layered sensory nature of biological skin.

Meanwhile, research into artificially reproducing pain sensation and with a view to application to robot skin has been gaining attention in recent years. These studies aim to detect excessive external stimuli as danger signals and link them to avoidance behavior and self-protection. In recent years, numerous approaches have been reported that focus on the nonlinear response and stimulus history-dependent characteristics of biological nociceptors and attempt to mimic these characteristics using artificial devices. For example, a system has been developed that uses diffusible memristors to achieve threshold responses, nonadaptation and sensitization to noxious stimuli in the same device, operating as an artificial nociceptor [8,9,10]. These approaches attempt to achieve information processing similar to that of the biological pain system by reproducing response changes depending on stimulus intensity and history [11,12,13]. However, from a practical engineering perspective, these memristor-based systems often face challenges regarding scalability and implementation complexity when applied to whole-body robot skin. Their reliance on specific material properties and complex hardware integration makes it difficult to adapt to diverse sensor configurations or to integrate high-level spatio-temporal processing. Consequently, there is a clear need for a software-driven framework that can realize these biological sensory functions through flexible algorithmic learning rather than fixed hardware characteristics.

Furthermore, considering that robots are intended for long-term operation in real-world environments, maintaining the integrity of the robot skin itself—which handles contact with the external world—is a critical challenge from the perspectives of safety and reliability [14]. In particular, failure to detect damage to the skin early can lead to degraded touch information and misrecognition, potentially resulting in inappropriate actions and reduced safety. Therefore, there is a need to establish damage detection technology where robots continuously monitor their own skin condition and detect deviations from normal states. A key point here is that damage does not necessarily manifest as clear breaks or missing parts; it often progresses gradually as minute degradation or localized changes in properties. Consequently, rather than predefining all possible damage forms, a framework that identifies abnormalities as deviations from the normal state is considered effective. On the other hand, existing research on damage detection for artificial skin often employs designs that directly treat responses to strong stimuli or physical property changes as damage indicators [15,16,17]. Critically, these approaches suffer from a logical conflation of stimulus intensity with structural integrity. By lacking a hierarchical sensory logic to distinguish between transient noxious contact and irreversible degradation, such systems are inherently prone to a high rate of false positives and excessive avoidance behaviors in practical robot operation.

To address these fundamental limitations of conventional methods, this study proposes a bio-inspired framework that acquires tactile stimuli, pain as a warning, and actual damage as distinct, hierarchical internal representations. Unlike existing research that conflates stimulus intensity with structural integrity [15,16,17], our approach is supported by neuroscience research indicating that pain is a predictive warning signal that forecasts future damage risk rather than a mere reflection of intensity [18]. Based on this perspective, we construct an artificial skin model using normal touch data as the sole input to define tactile, pain, and damage sensations as separate internal representations. Specifically, we implement an unsupervised anomaly detection model combining a Convolutional Autoencoder (CAE) and a Convolutional LSTM (ConvLSTM) to extract latent spatio-temporal features [19,20]. The main contributions of this study are summarized as follows:

1. Unsupervised representation learning from exclusively normal tactile data: By training the CAE-ConvLSTM architecture solely on touch maps of intact skin, the system learns to recognize “normal” spatio-temporal structures. This allows for the self-diagnosis of abnormalities—defined as pain or damage—without requiring explicit labels or a dataset of actual failure cases.
2. A bio-inspired hierarchical sensory framework: We propose a model that functionally dissociates tactile sensation, pain, and damage as distinct internal representations. This predictive hierarchy mimics biological logic, where pain acts as a warning signal for potential damage risk, rather than a mere reflection of raw stimulus intensity.
3. Significant reduction in false positives (FP) via pain-gated detection: By introducing a “pain-flag gating” mechanism—where damage is only assessed when a persistent structural deviation is preceded by a pain trigger—we minimize misdetections caused by momentary strong stimuli. This logically overcomes the inherent instability of conventional intensity-based thresholding methods.
4. Scalable and maintainable software-driven architecture: Unlike recent hardware-dependent neuromorphic or memristor-based systems, our software-centric approach ensures superior flexibility. This allows for easier sensor replacement, expansion, and adaptation to diverse robot configurations without the need for specialized hardware components.

## 2. Materials and Methods

This section describes the artificial skin used in the experiment, the anomaly detection model, and the overall system.

### 2.1. Artificial Skin

**Figure 1:**
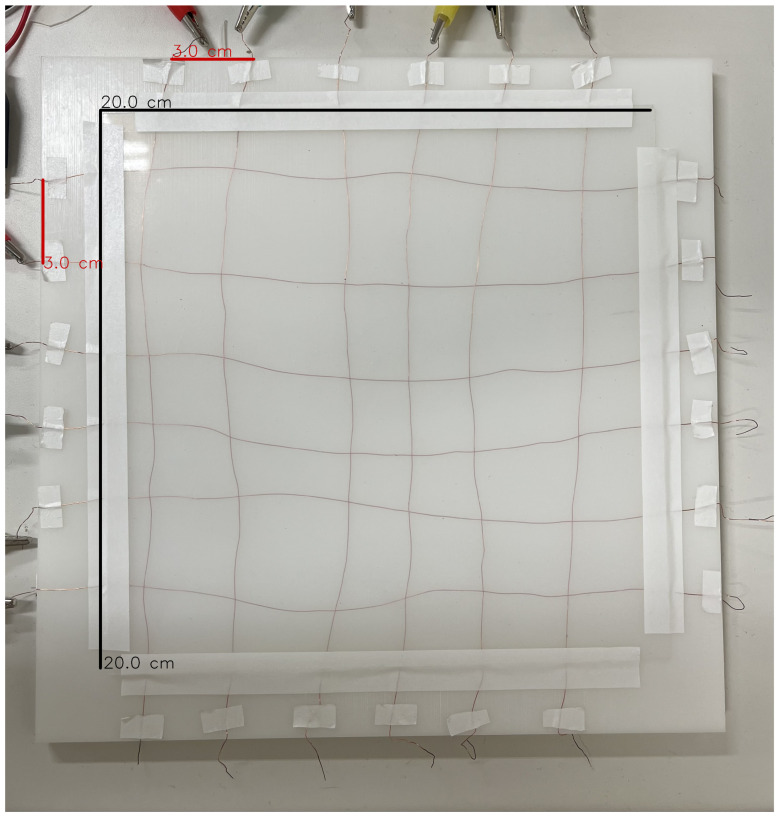
Artificial skin created for experiments. The red and black lines represent the scale bars for electrode spacing (3.0 cm) and substrate dimensions (20.0 cm), respectively. Due to geometric distortion during imaging, slight anisotropy (approximately 1–2%) occurred in the pixel scale; therefore, scale bars have been added with anisotropy corrected separately for the x- and y-axes based on known lengths.

The artificial skin developed in this study is a sensing system centered around MPR121 capacitive touch sensor controller (Adafruit Industries, New York, NY, USA). The MPR121 measures capacitance by detecting changes in the surrounding electric field distribution of each electrode, enabling the numerical acquisition of effects such as external pressure, proximity of conductive materials, and material deformation. Electrodes use UEW (urethane-coated wire) copper wire with an outer diameter of 0.26 mm (Kyowa Harmonet Ltd., Kyoto, Japan); the polyurethane coating serves as electrical insulation. Arranged in a grid pattern of six vertical and six horizontal wires on an insulating plate, they form 36 intersections. The UEW copper wire’s insulating coating not only prevents short circuits between electrodes but also functions as a dielectric layer. This structure facilitates the reflection of minute indentations and deformations in the electric field distribution. The crossing points of the copper wires were not electrically connected; instead, the upper and lower layers of the grid were wired independently. The distance between electrodes was set to approximately 3 cm. Each copper wire was lightly fixed only at the substrate edge using masking tape, allowing the central portion to flex freely. Limiting the number of fixed points in this way enables the electrodes to conform to localized indentations and deformations, yielding flexible response characteristics for the artificial skin as a whole. Furthermore, intentionally leaving minor placement irregularities recreates conditions closer to sensor individuality and operational errors in real environments, enabling the anomaly detection model to adapt to more practical conditions. Additionally, covering the entire electrode with a 1 mm thick silicone rubber sheet (Rimikuru, Trade Leading LLC, Hayama, Kanagawa, Japan) achieves a structure that disperses external forces while maintaining flexibility as an artificial skin. Silicone rubber possesses stable dielectric properties and flexibility. Placing it uniformly over the electrodes facilitates the reflection of indentation depth and spreading as capacitance changes. Figure 1 shows the actual fabricated artificial skin.

### 2.2. Dataset

This study constructed a dataset for unsupervised learning with the objective of learning the touch response of artificial skin in its normal state. The anomaly detection model described in the following section is designed to learn using only data from the normal state, detecting touch distributions and temporal changes not observed during training as anomalies. Therefore, it is crucial to strictly maintain the absence of damage to the artificial skin when acquiring training data.This study defined the normal state as one where the artificial skin has no clear damage such as cuts. Furthermore, touch actions simulating everyday contact with the artificial skin surface were defined as the touch response in the normal state.To construct the training dataset, the artificial skin described in the previous section was used, and the change in capacitance between electrodes was acquired as time-series data. The sensor output was acquired as digital values via the MPR121. The sensor values at all electrode intersections formed by arranging copper wire electrodes in a grid pattern vertically (H=6) and horizontally (W=6) were calculated and formatted into a 2D array of H×W. This array was defined as one frame of the touch map, and a sequence of 32 consecutive frames of touch maps was treated as one time-series data set.

While it is desirable to collect a wide range of contact data during experiments, the structure of artificial skin makes it difficult to reproduce all touches simulating everyday contact. Therefore, this study acquired touch data using only one hand. By varying the contact location while using the same hand, we generated tactile stimuli with different contact areas and pressure distributions. Furthermore, by varying the contact position across the entire artificial skin, a dataset comprising 300 data points was constructed, encompassing spatially diverse touch patterns. Furthermore, touch actions were not limited to fixed patterns; actions such as contact, pressing, sliding, and release were performed in random order and at random speeds. This enabled the acquisition of data containing temporally varying touch changes, allowing the model to learn variations in contact strength and temporal progression as general normal behavior. This aims to suppress excessive anomaly detection in real environments by training the model to recognize fluctuations caused by environmental noise or human proximity as normal states.

### 2.3. Outline of the Proposed Anomaly Detection Model

The proposed anomaly detection model was implemented using Python (Python Software Foundation, Wilmington, DE, USA). Deep learning architectures were constructed using the PyTorch library (Meta Platforms, Inc., Menlo Park, CA, USA), while scikit-learn, NumPy, and Matplotlib were employed for data preprocessing, statistical analysis, and visualization.

The input data is a touch map formed by arranging the 36 capacitive sensor values of the artificial skin shown in Figure 1 into a 6×6 two-dimensional grid, treated as time-series data of fixed length. The input *X* to the model is constructed as follows:

(1) $$X\in R^{B\times T\times 1\times 6\times 6},$$

where *B* denotes the batch size and *T* denotes the time series length.

This input *X* is passed through the CAE Encoder to extract spatial features, converting the touch map at each time point into a low-dimensional latent representation $Z_{t}$. The Encoder reduces the spatial resolution from 6×6 to 3×3 using a convolutional layer (kernel size 3, stride 2) from 1 to 4 input channels, and then expands the feature representation using a convolutional layer from 4 to 8 channels. Batch Normalization and LeakyReLU are applied after each convolutional layer to stabilize learning and suppress gradient vanishing. Dropout is introduced at the final stage of the Encoder to improve generalization performance against sensor variations and noise. The transformation by the Encoder is expressed as following equation:

(2) $$Z_{t}=f_{Enc}\left(X_{t}\right),$$

where $X_{t}$ is denoted as *X* at time *t*. The output $Z_{t}$ is constructed as follows:

(3) $$Z_{t}\in R^{B\times T\times 8\times 3\times 3}.$$

This $Z_{t}$ represents a compressed latent representation that preserves the spatial structure of the touch distribution. Inputting this $Z_{t}$ into the spatial decoder performs spatial reconstruction. The spatial decoder restores the resolution from 3×3 to 6×6 using a transposed convolutional layer, ultimately reconstructing a single-channel touch map $\hat{X_{t}}$. The output layer employs a sigmoid function, establishing correspondence with the normalized sensor values. The transformation by the Spatial Decoder is expressed as following equation:

(4) $${\hat{X}}_{t}^{recon}=f_{SpaceDec}\left(Z_{t}\right).$$

The difference between the input data *X* and the reconstructed result ${\hat{X}}_{t}^{recon}$ is calculated as the reconstruction error using the following equation:

(5) $$E_{t}^{recon}=\left|X_{t}-{\hat{X}}_{t}^{recon}\right|.$$

When an input *X* deviating from the touch distribution learned under normal conditions is applied, the reconstruction error $E_{t}^{recon}$ increases. This serves as an indicator for detecting spatial anomalies such as scratches, changes in electrode structure, or localized abnormal contact.

Next, for detecting temporal anomalies, the latent representations $Z_{t}$ are processed by a ConvLSTM with 64 hidden channels. In ConvLSTM, the hidden state $h_{t}$ and cell state $c_{t}$ are updated at each time step *t*. The cell state $c_{t}$ retains long-term temporal information accumulated from past input sequences, while the hidden state $h_{t}$ holds the output representation computed at time step *t* based on the cell state $c_{t}$. In this study, we adopt a ConvLSTM with a 64-channel hidden state, where both the hidden state $h_{t}$ and the cell state $c_{t}$ are initialized while maintaining a spatial size of 3×3. The update equations and dimensions of the hidden state $h_{t}$ and the cell state $c_{t}$ are expressed as follows:

(6) $$\left(h_{t+1},c_{t+1}\right)=f_{ConvLSTM}\left(h_{t},c_{t}\right)\;\left(h_{t},c_{t}\in R^{64\times 3\times 3}\right).$$

The ConvLSTM learns temporal patterns by updating its internal states with past touch sequences. To ensure pure predictions based solely on learned normal dynamics, the average normal touch map $\bar{X}$ is used as input for future time steps, preventing the model from incorporating temporary anomalies into its internal representation. The resulting hidden states are then processed by a temporal decoder—comprising transposed convolutional layers, Batch Normalization, and Dropout—to output the predicted touch map ${\hat{X}}_{t}^{pred}$ via a Sigmoid function. The transformation by the temporal decoder is expressed by the following equation:

(7) $${\hat{X}}_{t}^{pred}=f_{TimeDec}\left(h_{t}\right).$$

The difference between the input data $X_{t}$ and the predicted result ${\hat{X}}_{t}^{pred}$ is calculated as the prediction error using the following equation:

(8) $$E_{t}^{pred}=\left|X_{t}-{\hat{X}}_{t}^{pred}\right|.$$

The prediction error $E_{t}^{pred}$ serves as an indicator reflecting temporal structure abnormalities, such as touch response irregularities caused by damage.

### 2.4. The Definitions of Touch, Pain and Damage

**Figure 2:**
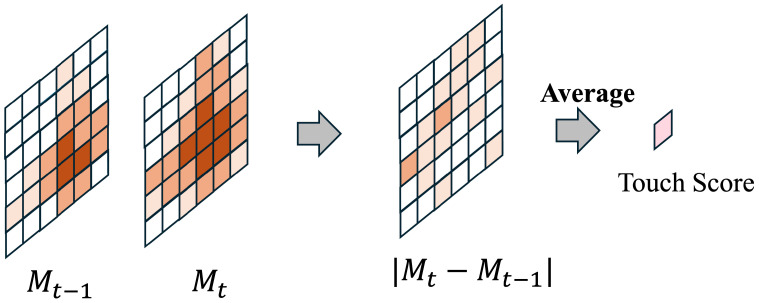
Calculation process of the touch score.The score is derived by calculating the absolute difference between consecutive frames of touch maps ($M_{t-1}$ and $M_{t}$) and then computing the average of these differences across the entire grid.

In this study, we define touch as the function of detecting changes in sensor signals generated by physical contact or proximity input to artificial skin. Touch is a low-level sense that first detects the occurrence of interaction with the external environment, serving to detect the presence or absence of stimuli and the occurrence of changes themselves. In human skin, tactile sensation perceives contact with the external environment by receiving stimuli such as pressure, vibration, and shear. However, the signals themselves do not necessarily directly indicate danger or damage. Similarly, the touch function of the artificial skin in this study is positioned as a fundamental indicator for detecting transitions to a contact state, serving as a preliminary step before evaluating the intensity or abnormality of the stimulus. This study focuses on the temporal variation in touch data obtained from capacitive sensors, defining touch by using signal differences between consecutive frames. Figure 2 illustrates how the touch score is calculated.

The absolute value of the difference between the touch map $M_{t}$ at time t and the touch map $M_{t-1}$ at time t−1 is calculated, and its average value is used as the touch data. In a non-contact state, only fluctuations due to environmental noise and minute variations are observed, whereas when contact occurs, a temporal change exceeding these fluctuations occurs. Utilizing this property, contact detection is performed based on the statistical distribution of the temporal change amount in the non-contact state. In this study, the metric calculated based on this temporal change is termed the touch score. When the touch score exceeds a predetermined threshold, the artificial skin is judged to have entered a contact state. The touch score does not directly indicate the danger of a stimulus or the presence of damage; it is solely an indicator for determining whether interaction with the external environment has occurred.

In this study, we define pain perception in artificial skin as an internal metric representing the degree of instantaneous deviation from a model trained with a touch map as the normal state. Recognizing that biological pain perception is an information processing mechanism for detecting signs of abnormality or damage threatening bodily homeostasis, rather than the physical magnitude of the stimulus itself, we also position pain perception in this study as an abstract signal representing the intensity of abnormality.

**Figure 3:**
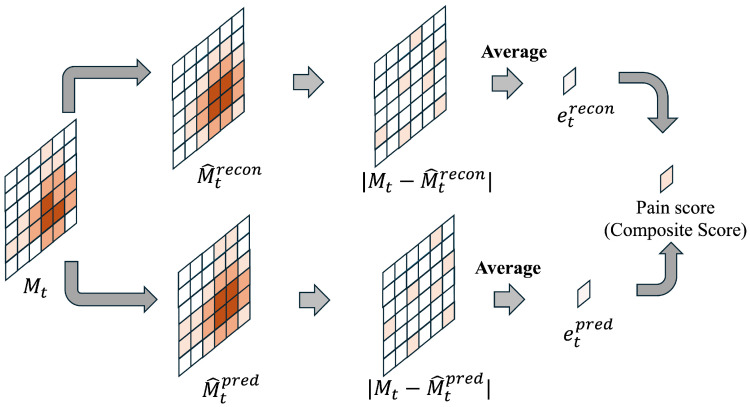
Calculation process of the pain (composite) score using the reconstruction error and the prediction errors.The process integrates spatial reconstruction errors ($|M_{t}-{\hat{M}}_{t}^{recon}|$) and temporal prediction errors ($|M_{t}-{\hat{M}}_{t}^{pred}|$) into a pain (composite) score.

The artificial skin studied in this research acquires touch maps as time series data from 36 capacitive sensors. For these touch maps, the spatial and temporal normal structures are modeled using the CAE and ConvLSTM described in the previous section. In the normal state, the touch distribution is reconstructed and predicted with high accuracy by the trained model. However, when damage, changes in electrode structure, or abnormal contact occur, the structure deviates from the model’s assumptions, leading to increased reconstruction error and prediction error. These represent abnormal intensities of sensory input. Therefore, this study defines a quantity integrating these two types of error as the pain score. Figure 3 illustrates how the pain score is calculated. Thus defined, the pain score is an abstract internal signal independent of specific contact locations or stimulus directions, functioning as an indicator for artificial skin to self-diagnose its own state.

**Figure 4:**
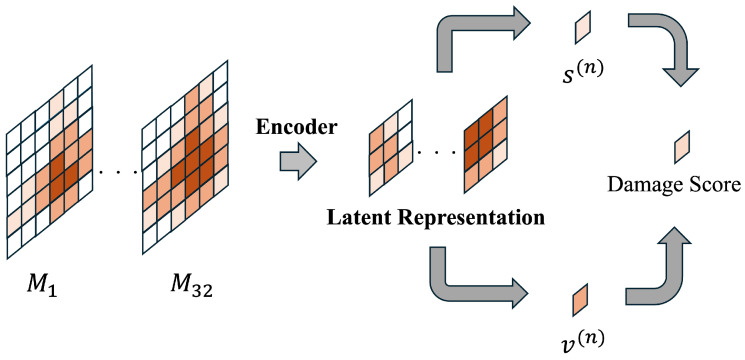
Calculation process of the damage score based on latent representations.The damage score is derived by integrating the structural component ($s^{\left(n\right)}$) and the temporal persistence component ($v^{\left(n\right)}$) from the latent feature maps.

In this study, we define damage as a state where the spatial distribution of touch persistently deviates from the characteristics learned as the normal state, resulting from structural changes occurring in the artificial skin. Therefore, the damage score is calculated by focusing on persistence from a latent representation map containing structural information. Figure 4 illustrates how the damage score is obtained.

Thus, the damage score is designed to increase when errors are concentrated locally or when abnormalities are observed persistently rather than briefly. In other words, while using the same touch information, pain perception and damage are differentiated and designed with distinct purposes.

### 2.5. Learning Process

**Figure 5:**
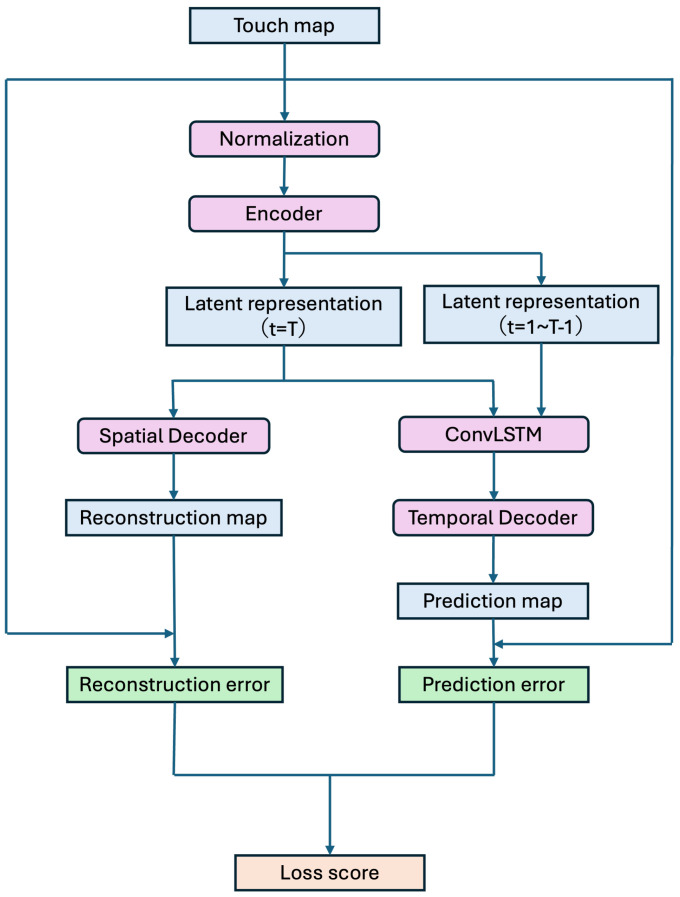
Learning process for a single time-series sequence.

Figure 5 illustrates the learning process for a single item of dataset.

In this study, we pre-train the model on touch responses from normal state using the dataset constructed in the previous section. First, for each training data point, we perform spatial reconstruction using CAE on the last frame of a sequence of 32 consecutive frames. The touch map $X_{t}$ at time t=32 is input to the CAE encoder, which compresses it into a low-dimensional latent representation $Z_{t}$ that preserves the spatial structure of the sensor distribution. This latent representation $Z_{t}$ is passed through a spatial Decoder to output a reconstructed touch map ${\hat{X}}_{t}^{recon}$ similar to the input. The reconstruction loss $L_{recon}$ is defined as the difference between this touch map $X_{t}$ and the reconstructed touch map ${\hat{X}}_{t}^{recon}$. This study adopts the mean absolute error (L1 Loss) because it is robust to outliers and can stably evaluate local errors. The reconstruction loss $L_{recon}$ is expressed by the following equation:

(9) $$L_{recon}=\frac{1}{HW}\sum_{i,j}\left|\right|X_{t}\left(i,j\right)-{\hat{X}}_{t}^{recon}\left(i,j\right)\left|\right|.$$

Here, H=W=6. This loss enables CAE to learn to stably reconstruct the input while preserving the spatial structure of the touch distribution in the normal state.

Next, we perform temporal prediction using ConvLSTM. We adopt a configuration where the touch state at the 32nd frame is predicted based on the touch information from the previous 31 frames within a sequence of 32 frames from the training data. The prediction loss $L_{pred}$ is defined as the difference between the predicted touch map $X_{t}$ at time *t* and the difference between the predicted touch map ${\hat{X}}_{t}^{pred}$ generated from the latent representation sequence $Z_{t}$ obtained by the CAE Encoder and the latent representation sequence from past touch sequences at times t−31 to t−1. The prediction loss $L_{pred}$ is similarly defined using L1 Loss, expressed by the following equation:

(10) $$L_{pred}=\frac{1}{HW}\sum_{i,j}\left|\right|X_{t}\left(i,j\right)-{\hat{X}}_{t}^{pred}\left(i,j\right)\left|\right|.$$

Here, H=W=6. This loss enables ConvLSTM to learn the temporal variation patterns of normal touch responses as internal states, allowing it to stably predict future touch distributions.

In this study, to construct an anomaly detection model sensitive to both spatial and temporal anomalies, we perform learning using a composite loss score that integrates reconstruction loss and prediction loss. The composite loss score $L_{comp}$ is defined as following equation:

(11) $$L_{comp}=\frac{L_{recon}+\lambda L_{pred}}{1+\lambda }.$$

Here, λ is the weighting coefficient that adjusts the contribution of the reconstruction error and prediction error. In this study, λ=0.8. It reflects the prioritized weighting of temporal prediction (ConvLSTM) against spatial reconstruction (CAE). By setting λ=0.8, the model is tuned to prioritize spatial reconstruction slightly less than temporal dynamics, ensuring the system remains acutely sensitive to the progression of damage over time rather than just instantaneous spatial anomalies.

When training, perform backpropagation of errors across the entire model using this composite loss. Specifically, optimize all parameters θ—including the CAE’s Encoder, spatial Decoder, ConvLSTM, and temporal Decoder—using the following approach:

(12) $$\theta \leftarrow Adam(\theta ,\nabla_{\theta }.L_{comp}\eta ).$$

Here, η is the learning rate. In this study, the Adam optimizer is used to achieve stable convergence. In this study, $\eta =1.0\times 10^{-3}$.

### 2.6. Pain Score Distribution and Damage Score Distribution

This section explains how to calculate the statistical measures for the pain score and damage score based on the normal state, which are required for the real-time detection described in the next section.

As explained in the previous section, the pain sensation in this study is an internal metric representing the degree of instantaneous deviation from the spatial and temporal structure of the normal touch map. It is defined not by a single stimulus intensity but as a combination of error structures. The composite score used for learning precisely reflects this spatial and temporal deviation in the touch distribution. Therefore, we matched the distribution of the composite score to the normal distribution of the pain score. We then set the pain threshold at the upper 95% of this normal distribution.

As explained in the previous section, the damage score is designed not as a measure of instantaneous error magnitude, but as an indicator quantifying persistent structural deviation. In this study, damage is treated as an anomaly in the latent representation of touch distributions. We calculate structural components and temporal persistence components from the latent feature maps obtained by the CAE Encoder, and use these to define the damage score. First, we apply the Encoder to the input touch map of a single data point (a sequence of *T* frames) to obtain the latent sequence $Z^{\left(n\right)}$ as follows:

$$Z^{\left(n\right)}=\left\{Z_{1}^{\left(n\right)},Z_{2}^{\left(n\right)},\ldots ,Z_{T}^{\left(n\right)}\right\}\;\left(Z^{\left(n\right)}\in R^{C\times H\times W}\right).$$

Here, *n* represents the training data number, and *C* denotes the number of channels in the latent representation. In this study, C=8, H=W=3, and T=32. We compute this latent sequence $Z^{\left(n\right)}$ for all training data and calculate the entire latent sequence *Z* as follows:

$$Z=\left\{Z^{\left(1\right)},Z^{\left(2\right)},\ldots ,Z^{\left(N\right)}\right\}\;\left(Z\in R^{N\times T\times C\times H\times W}\right).$$

In this study, N=300. From this entire latent sequence *Z*, the latent mean μ required for the learning process is obtained. First, the temporal mean $M_{time}^{\left(n\right)}$ of the latent sequence $Z^{\left(n\right)}$ is calculated as follows:

(13) $$M_{time}^{\left(n\right)}=\frac{1}{T}\sum_{t=1}^{T}Z_{t}^{\left(n\right)}\;\left(Z_{t}^{\left(n\right)}\in R^{C\times H\times W}\right).$$

Next, by taking the data-weighted average of the time-averaged $M_{time}^{\left(n\right)}$, the latent mean μ is calculated as follows:

(14) $$\mu =\frac{1}{N}\sum_{n=1}^{N}M_{time}^{\left(n\right)}\;\left(\mu \in R^{C\times H\times W}\right).$$

The structural component represents an indicator emphasizing changes in the spatial structure of the touch distribution itself, such as alterations in electrode placement or damage to skin layers. In this study, the absolute value of the difference between the latent representation $Z_{T}^{\left(n\right)}$ of the latest frame and the normal latent mean μ is used to calculate the structural component $s^{\left(n\right)}$ using the following equation.

(15) $$s^{\left(n\right)}=\frac{1}{CHW}\sum_{c,h,w}\left|Z_{T}^{\left(n\right)}\left(c,h,w\right)-\mu \left(c,h,w\right)\right|,$$

where $Z_{T}^{\left(n\right)}\left(c,h,w\right)$ is a scalar representing the (c,h,w) element of $Z_{T}^{\left(n\right)}$. Next, the temporal persistence component represents an indicator that suppresses the misdetection of fluctuations in latent representations caused by momentary strong contacts or noise as damage. First, using the temporal average $S_{time}^{\left(n\right)}$ of the latent sequence, the difference $\Delta Z_{t}^{\left(n\right)}$ between the latent representation $Z_{t}^{\left(n\right)}$ at each time point and the temporal average $S_{time}^{\left(n\right)}$ is calculated as follows:

(16) $$\Delta Z_{t}^{\left(n\right)}=Z_{t}^{\left(n\right)}-S_{time}^{\left(n\right)}\;\left(\Delta Z_{t}^{\left(n\right)}\in R^{C\times H\times W}\right).$$

Using this metric, the spatial norm $V_{t}^{\left(n\right)}$ at each time *t* for the nth data point is calculated as follows:

(17) $$V_{t}^{\left(n\right)}=\sqrt{\frac{1}{CHW}\sum_{h,w}{\left(\frac{1}{C}\sum_{c}\Delta Z_{t}^{\left(n\right)}\left(c,h,w\right)\right)}^{2}}.$$

Here, $\Delta Z_{t}^{\left(n\right)}\left(c,h,w\right)$ denotes the scalar representing the (c,h,w) element of $\Delta Z_{t}^{\left(n\right)}$. While each latent channel carries distinct feature representations, our temporal stability evaluation prioritizes temporal variations in spatial distribution over inter-channel differences. Therefore, we employ representations averaged across channel dimensions to suppress channel-specific influences. Furthermore, to prevent the score scale from varying due to differences in the dimensionality of the latent representations or the number of channels, this study evaluates using the average energy normalized by the number of channels and the spatial size. Subsequently, the temporal persistent component $v^{\left(n\right)}$, which suppresses short-term fluctuations, is obtained by time-averaging this spatial norm as follows:

(18) $$v^{\left(n\right)}=\frac{1}{T}\sum_{t=1}^{T}V_{t}^{\left(n\right)}.$$

The final damage score $d^{\left(n\right)}$ is obtained by integrating the structural component and the temporal persistence component with weights as follows:

(19) $$d^{\left(n\right)}=\alpha s^{\left(n\right)}+\left(1-\alpha \right)v^{\left(n\right)}.$$

Here, α is a parameter that adjusts the contribution of both components, and in this study, α=0.8. In calculating the damage score, the structural component $s^{\left(n\right)}$, which represents the deviation from the baseline state in latent space, was given priority over the temporal variation component $v^{\left(n\right)}$. This is to ensure that irreversible changes, such as deep incisions, are reliably captured. During the learning step, only normal-state data is used to estimate the distribution of damage scores, which is then employed as a statistical reference. The pain threshold was set at the upper 95% of the normal distribution of composite scores obtained during the training phase. This value was selected as a statistically significant criterion for identifying outliers that deviate from the learned normal spatio-temporal structure. Furthermore, the damage detection employs a two-stage threshold—95% for activation and 90% for deactivation. This hysteresis mechanism is intentionally designed to suppress false detections caused by momentary signal fluctuations or environmental noise, ensuring a stable transition to the damaged state only when persistent structural deviations are present.

### 2.7. Real-Time Anomaly Detection

**Figure 6:**
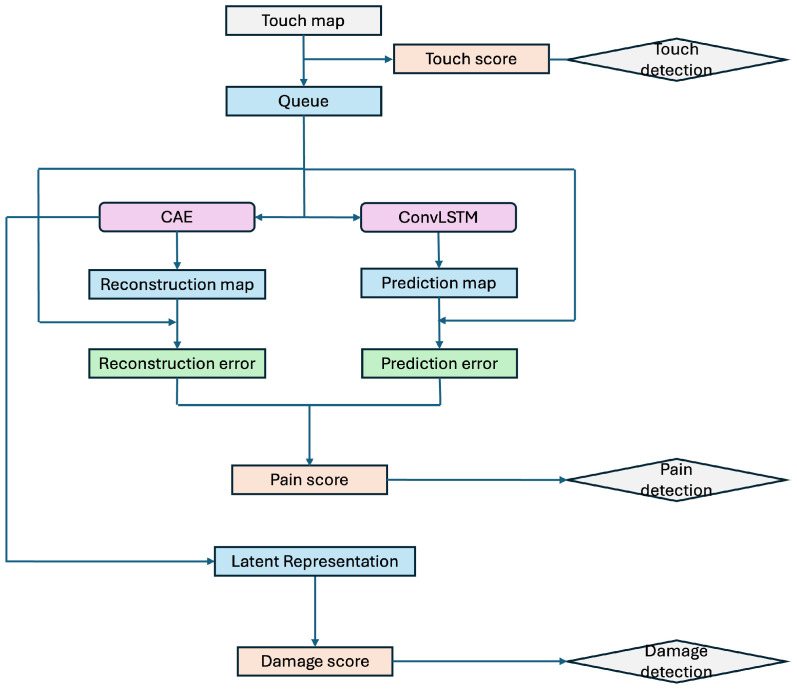
The procedure from acquiring a single frame of the touch map to performing each determination.

Figure 6 shows the procedure from acquiring a single frame of the touch map to performing each determination.

The capacitive sensor of the artificial skin created in the previous section formats the sensor values acquired at each time point into a 6×6 grid-like touch map. The touch map is sequentially transmitted to the PC via serial communication through an Arduino Uno microcontroller (Arduino SA, Monza, Italy) and the MPR121 for real-time processing. After system startup, a calibration phase is first established to confirm the artificial skin is in an unloaded and undamaged state. After calibration completes, the touch maps received from the sensors are sequentially accumulated in a time-series buffer. First, the difference between the current touch map and the touch map from one frame prior is calculated for each consecutive frame. The average of the absolute values of these differences is recorded as the touch score for one sample. This process is repeated for 100 frames to obtain the distribution of touch scores in the non-contact state. The mean and variance are calculated for the obtained 100 samples, and the touch threshold is determined based on these values. In this study, to suppress the effects of sensor noise and minor environmental fluctuations, the upper and lower hysteresis thresholds are set using the mean and variance obtained during the non-contact state. After acquiring the touch score distribution, the real-time detection loop begins. The touch map $M_{t}$ is obtained from the artificial skin, the touch score is calculated, compared against the touch threshold, and the touch flag is updated. Additionally, the touch map $M_{t}$ is input into a queue of length T = 32 to form time-series data, which is then fed into a pre-trained anomaly detection model. This model processes the data to calculate the pain score (composite score) and damage score defined in the previous section. These are compared against the pain threshold and damage threshold determined during training, updating the pain flag and damage flag accordingly.

Additionally, in real-time detection, we introduced a dynamic pain threshold function and a pain flag gating function that mimic living skin. The pain threshold $\theta_{t}$ at each time step used in the dynamic pain threshold function is calculated by subtracting the variable $\delta_{t}$—representing the internal state obtained in the previous detection loop—from the baseline value *b* obtained during the learning phase, as shown in the following equation:

(20) $$\theta_{t}=clip\left(b-\delta_{t},\theta_{min},\theta_{max}\right).$$

Here, $\delta_{min}$ and $\delta_{max}$ represent the lower and upper limits of the threshold. Additionally, to prevent false detections caused by threshold exceedances in a single frame, this study retains the judgment results for the most recent two frames. The pain flag is established only when all of these results are true. Subsequently, the internal state $\delta_{t}$ used in the next detection loop is updated. The process where the pain flag increases the internal state $\delta_{t}$, thereby lowering the threshold $\theta_{t}$ for subsequent stimuli. When the pain flag is established, the internal state $\delta_{t}$ is increased as shown in the following equation:

(21) $$\theta_{t+1}=\delta_{t}+k\left(\delta_{max}-\delta_{t}\right).$$

The gradual normalization of the internal state when the pain flag is absent, representing the return to homeostasis. In this study, $\delta_{max}=0.1$ and k=0.5. By setting k=0.5 in the above equation, the model ensures a rapid reduction of the pain threshold $\theta_{t}$ upon the detection of noxious stimuli, effectively mimicking the biological sensitization process observed in living skin. This dynamic adjustment allows the system to become more hypersensitive to subsequent inputs, thereby enhancing its proactive self-protection capabilities against potential structural damage. On the other hand, when the pain flag is not set, the internal state $\delta_{t}$ is decreased as shown in the following equation:

(22) $$\theta_{t+1}=\delta_{t}+l\left(\delta_{min}-\delta_{t}\right).$$

In this study, $\delta_{min}=-0.1$ and l=0.005. By employing a significantly smaller value as *l* compared to the sensitization coefficient *k*, the model characterizes the gradual return to homeostasis observed in biological sensory systems. This mechanism ensures that the pain threshold $\theta_{t}$ restores incrementally to its baseline level once noxious stimuli cease, allowing the artificial skin to transition from a hypersensitive state back to its normal operational equilibrium in a stable manner. Furthermore, based on the finding that pain serves as a warning signal for potential damage in living organisms, we introduced damage detection gated by a pain flag as a pain flag gating function. Specifically, this method imposes the constraint that no new damage occurs when no pain is present.

### 2.8. Experiment Conditions and Procedure

**Figure 7:**
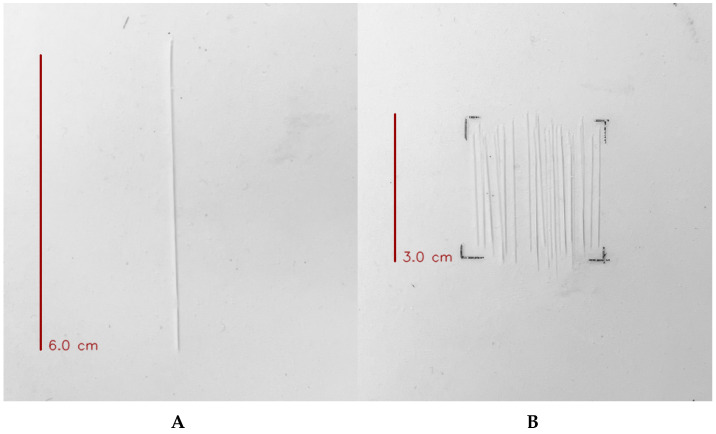
The image of the damage applied to the skin in the overall experiment. (**A**) The image when applying a cut (6 cm) to the skin; (**B**) The image when applying a abrasion (30 times) to the skin.

This study conducts experiments to determine whether cuts and abrasions—representative damage to artificial skin—can be detected. Cuts are defined as deep incisions made with a utility knife that penetrate the skin layer and reach the underlying electrode. The incision size is set to three conditions (3 cm, 4.5 cm, and 6 cm). Abrasions are defined as multiple shallow cuts made with a utility knife that do not penetrate the skin layer. The number of abrasions is set to three conditions (10 times, 20 times, and 30 times). This design allows for a stepwise evaluation of how the artificial skin model responds under varying degrees of damage. The following Figure 7 shows examples of cuts and abrasions actually created in the experiment.

To quantify these damage conditions, the number of touches after damage onset must be standardized. Therefore, this study introduced a mechanism allowing log recording during real-time detection to be initiated via the UI (user interface), which provides a means for users to control the system during operation. At each time point, various metrics—including scores and status flags—were recorded and saved in CSV format.

The experimental procedure is summarized as follows. This study involves three experimental items: (I) Applying strong touch to undamaged artificial skin to verify the presence or absence of false damage detection. (II) Applying weak touch to both undamaged and damaged artificial skin to confirm the behavior of each score and damage detection. (III) Applying uniform contact stimulation to artificial skin with a predetermined incision to measure the time required for damage detection. Furthermore, as a ablation experiment, classical threshold-based methods that apply a fixed threshold to raw sensor signals were set as the method, and quantitative metrics for both the proposed method and the baseline method were calculated using the data from Experiment (II). Additionally, hyperparameters were modified, and touch tests were conducted under the same conditions as in Experiment (II). Based on these results, we demonstrate the superiority of the proposed method.

## 3. Results

### 3.1. Experiment (I)

**Figure 8:**
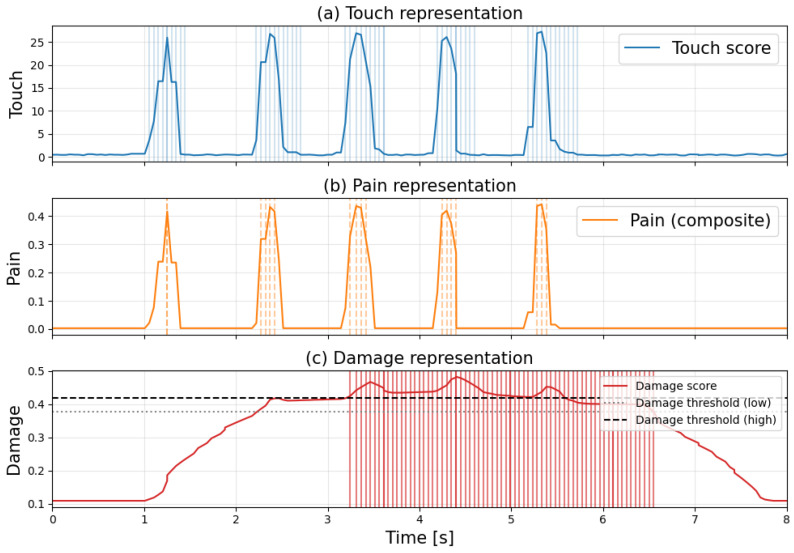
Time-series response to noxious stimuli on intact skin. (Each score is computed based on different definitions and normalization methods; therefore, their absolute values are not directly comparable. The figure focuses on the relative changes in each score over time).

Figure 8 shows the time-series graphs of each score when applying a strong touch to normal skin every second, with shaded areas indicating the detection times for each flag. Figure 8a–c show touch, pain, and damage information for the skin, respectively.

Figure 8 confirms that the touch flag and pain flag are established with each increase or decrease in the score. Meanwhile, it can be observed that the damage flag is established during certain intervals. The graph indicates that the damage score increases over time until it approaches the threshold. Upon interrupting the stimulus, the damage score gradually decreases, and after a certain period, the damage determination is lifted, restoring the system to its normal state. This behavior mimics the biological touch aftereffect, resulting from the temporal integration characteristics of the central nervous system and the response decay of mechanoreceptors. According to the experimental results in Figure 8, out of a total of 340 frames, damage was detected in 72 frames, resulting in a false positive rate of approximately 21%.

### 3.2. Experiment (II)

**Figure 9:**
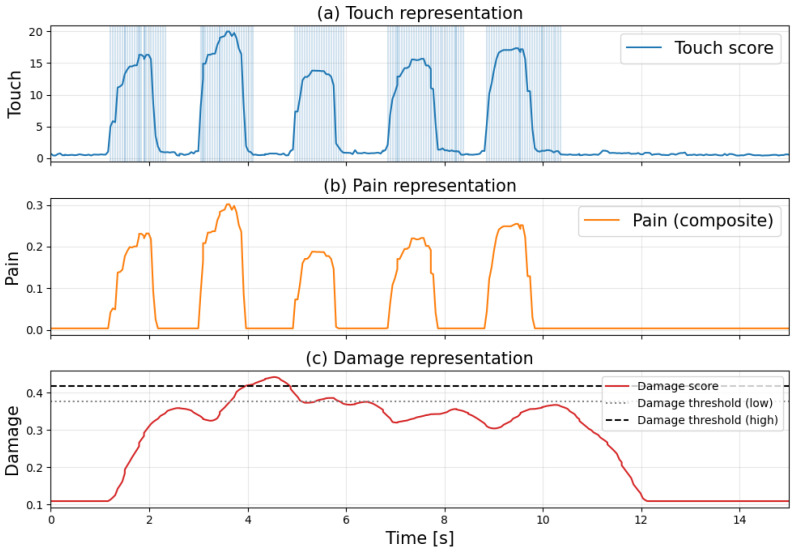
Time-series graphs of each score when touching normal skin and the times when each flag was established. (Each score is computed based on different definitions and normalization methods; therefore, their absolute values are not directly comparable. The figure focuses on the relative changes in each score over time).

**Figure 10:**
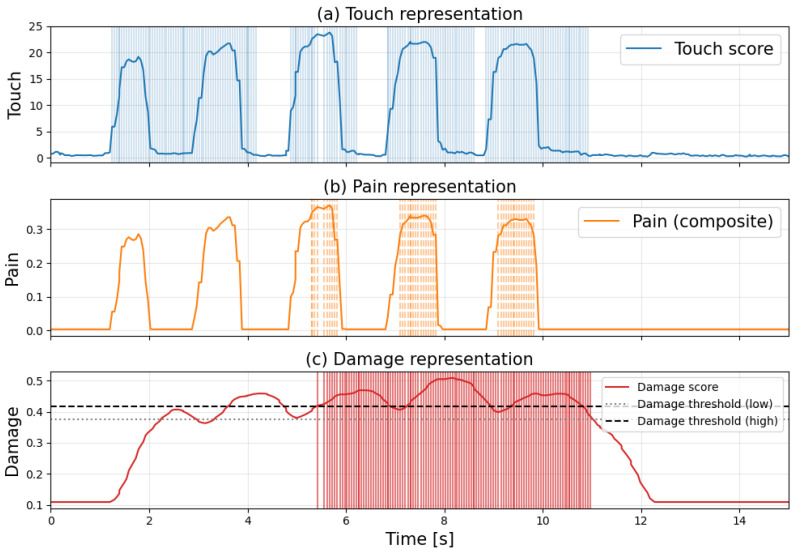
Time-series graphs of each score when touching the damaged skin and the times at which each flag was triggered. (Each score is computed based on different definitions and normalization methods; therefore, their absolute values are not directly comparable. The figure focuses on the relative changes in each score over time).

Figure 9 and Figure 10 show the time course of each score when applying the same intensity touch for 1 s followed by a 1-s rest interval to undamaged skin and damaged skin (3 cm cut). The shaded areas indicate the time each flag was established. Figure 9 and Figure 10a–c show touch, pain, and damage information for the skin, respectively.

Figure 9 and Figure 10 show that the distribution when no touch was applied became disordered due to the damage, and differences were observed in how each score fluctuated. First, in Figure 9, the damage score increased each time the sensor detected a touch, and moments were observed where it exceeded the upper damage threshold. However, it was found that damage determination did not occur because the pain flag was not set. Subsequent damage scores fluctuated but generally decreased, recovering when touch ceased. In contrast, Figure 10 shows that the magnitude of increases in each score was slightly larger compared to normal conditions. While the touch flag was set in response to increases and decreases in the touch score, there were intervals where the touch flag remained set even without touch. Regarding the pain flag, it was initially not set, but it was confirmed that it became set each time the pain score changed after a certain point in time. Meanwhile, the damage score exceeded the upper damage threshold faster than pain detection. When the pain flag was set, the damage flag also became set, maintaining the damage determination for a certain period. Subsequently, when the pain flag disappeared, the damage score decreased and recovered.

### 3.3. Experiment (III)

**Figure 11:**
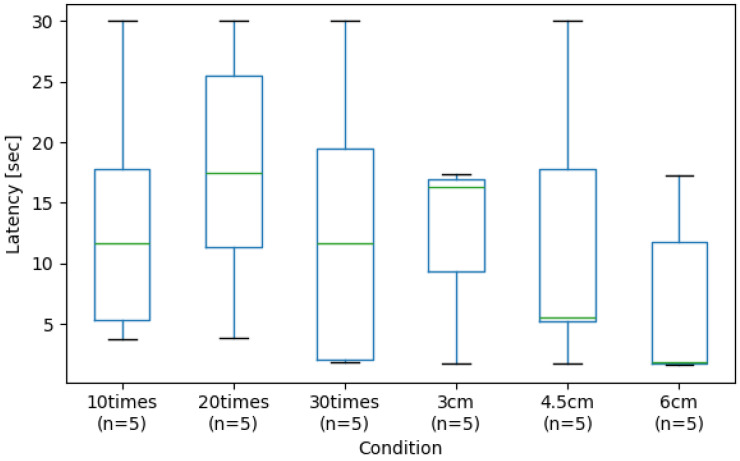
Latency of damage detection across experimental conditions. (*n* denotes the sample size for each condition, as indicated in the x-axis labels).

Figure 11 shows the interquartile range of the time variation required to detect damage when touching the skin at intervals of one second on and one second off, with a maximum duration of 30 s, for each incision (3 cm, 4.5 cm, 6 cm) and each abrasion (10 times, 20 times, 30 times). The green line indicates the median.

Figure 11 shows that for abrasions, no regularity in detection time was observed with increasing number of trials, and the results exhibited significant variability. In contrast, comparing the median values across each incision condition revealed a trend where the time to detect damage decreased as the incision length increased. This phenomenon is attributed to the fact that larger lacerations induce more pronounced structural deviations in the latent representation space, making them more readily reflected in the damage score. Furthermore, the shortest detection time was nearly identical across all conditions, with low variability in the distribution. However, some trials under the 4.5 cm incision condition did not result in damage detection.

### 3.4. Quantitative Evaluation of the Proposed Model Using Ablation Experiments

In this section, results of quantitative evaluations and parameter ablation experiments are presented, in contrast to previous qualitative evaluations based on plots.

#### 3.4.1. Comparison the Proposed Method with the Baseline Method

The following table and figures show the confusion matrices, key metric values, and ROC curves for the proposed method and the baseline method. Note that for fair evaluation, the baseline’s threshold was adjusted to achieve a detection sensitivity equivalent to the recall of the proposed method.

**Figure 12:**
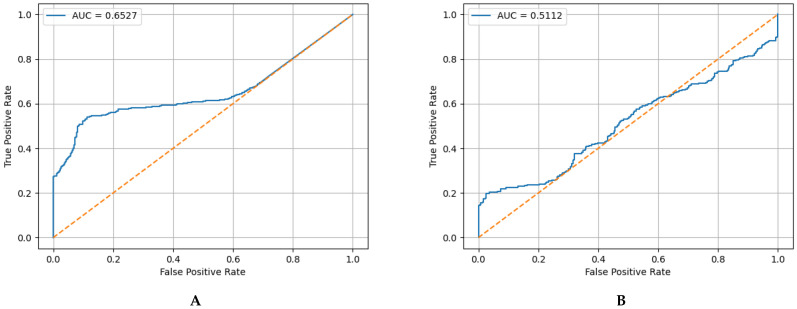
Comparison of the ROC curves and the AUCs between the proposed method and the baseline method. (**A**) The ROC curves and AUC value for the proposed method. (**B**) The ROC curves and AUC value for the baseline method.

Based on the Table 1, while the baseline method exhibited residual randomness in terms of accuracy and precision and generated a large number of false positives, the proposed method maintained extremely high reliability, with an accuracy of 0.6779, a precision of 1.0, and zero false positives. This quantitatively demonstrates that the proposed method does not confuse noise or strong transient stimuli with damage, as a simple thresholding method might, and clearly distinguishes between temporary anomalies and persistent structural changes. Based on the ROC curve for the baseline method shown in the Figure 12, the AUC is only 0.511. This value is very close to that of random classification, suggesting that a decision based solely on the intensity of the raw sensor signal cannot statistically distinguish between normal strong contact and structural damage. In contrast, the ROC curve for the proposed method in the Figure 12 shows an AUC of 0.6527, indicating that the proposed method does not rely on a single threshold setting and is capable of statistically separating normal and damaged states.

Furthermore, we demonstrate that the proposed method can reliably distinguish between the normal and damaged states. The following table shows the number of damage flag switches for the proposed method and the baseline method under normal and damaged conditions. Note that the measurement interval is from the time when the damage flag was first set until the end of the measurement.

Based on the Table 2, the baseline method was found to frequently switch damage flags in response to subtle fluctuations in sensor signals and sudden spikes during contact. In contrast, the proposed method significantly reduced the number of such switches, yielding extremely stable results.

#### 3.4.2. The Impact of Changing Hyperparameters on the Model

In this section, we quantitatively evaluate whether the hyperparameters (λ=0.8, α=0.8) are appropriate. The following table shows the confusion matrix and key metric value when λ and α are changed.

Based on the Table 3, when (λ,α)=(0.2,0.8), the number of false positives surged to 100. Although the precision and recall rates remained relatively high, the recall, F1 score, and AUC dropped significantly. Therefore, it was established that reliability of the damage detection system had been lost. Furthermore, when (λ,α)=(0.8,2.0), both true positives and false positives are zero, indicating that the model is not making correct classifications. Meanwhile, when (λ,α)=(2.0,0.8),(0.8,0.2), the number of false positives is zero, and the values of the key metrics appear to outperform those of the proposed method. The following figure shows the time-series graphs of each score under normal conditions when (λ,α)=(2.0,0.8),(0.8,0.2), along with the times at which each flag was set.

**Figure 13:**
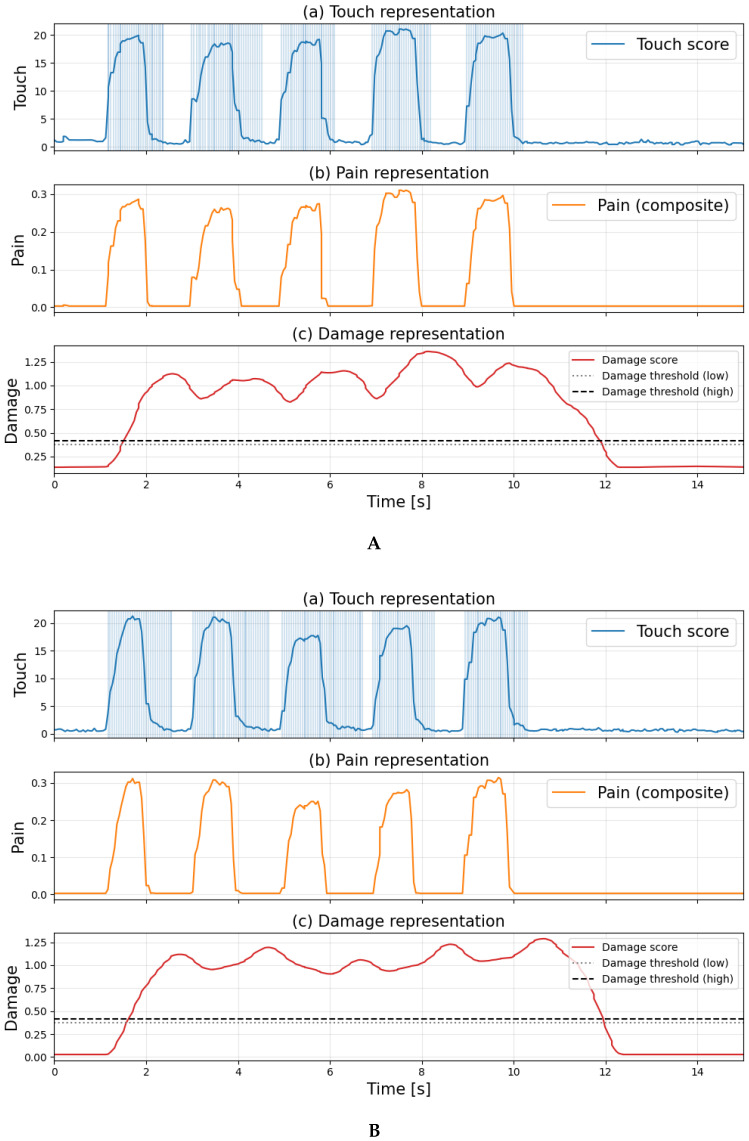
Model performance when changing hyperparameters. (**A**) Time-series graphs of each score when touching normal skin and the times at which each flag was triggered, when (λ,α)=(2.0,0.8). (**B**) Time-series graphs of each score when touching normal skin and the times at which each flag was triggered, when (λ,α)=(0.8,0.2).

As shown in the Figure 13, the damage score sharply increases upon touch and consistently exceeds the damage threshold. This indicates that, due to the pain gating function of the proposed method, the damage flag is set at the same time the pain flag is set. Therefore, when in a normal state, the system can be considered to be in an unstable condition from the perspective of damage detection.

Based on these results, the proposed values ((λ,α)=(0.8,0.8)) provide a valid setting that completely eliminates false positives while maintaining an optimal balance between classification stability and sensitivity.

## 4. Discussion

### 4.1. Regardinng the Experiment Results

This study proposes a framework for defining touch, pain, and damage in artificial skin based on touch data, and for separating and evaluating each as distinct scores. Experimental results confirmed that the proposed method exhibits different responses depending on differences in stimulus intensity and structural changes.

First, in Experiment (I), cases were observed where the damage flag was triggered even on normal skin in certain segments. This is thought to stem from the proposed model interpreting temporally close strong stimuli as sustained stimuli, leading to false damage detection. Importantly, the damage state was released after the interruption of the stimulus, indicating that the model does not treat damage as an irreversible state. This behavior is consistent with the design principle of representing damage as a recoverable condition depending on temporal context.

Experiment (II) revealed differences in how each score increased and decayed. This is thought to be caused by structural changes resulting from sensor disruption when damage occurred. Therefore, even when applying the same touch to normal skin and damaged skin, it is considered to have affected the scores for the damaged state. Additionally, intervals exceeding the upper damage threshold were observed in some damage scores for normal skin, yet the damage flag did not activate. This failure to establish the pain flag indicates that the damage determination gating mechanism based on pain detection is functioning. This signifies that the artificial skin reproduces behavior similar to human skin sensation: it does not classify states without pain as damage, even when internal structural disruption exists.

Additionally, intervals were observed where the touch flag remained active despite no touch occurring. This is thought to be caused by the contact distribution not completely disappearing due to sensor micro-fluctuations, residual charge, or the temporal smoothing processing of the touch map, leading the model to interpret the contact state as continuing. In other words, contact states may persist within the model regardless of actual damage presence. While this may appear as a touch misdetection, similar functions are observed in biological skin. For instance, even immediately after removing a hand from an object, the sensation of pressure or contact does not vanish instantly and may be perceived as a residual sensation. This is thought to result from the response characteristics of skin mechanoreceptors and temporal integration processing within the central nervous system. In our model, the interval where the tactile flag remains active corresponds to this biological touch aftereffect behavior. By not treating the contact state as an instantaneous binary judgment, this approach can be interpreted as achieving processing closer to that of biological skin.

In Experiment (III), no consistent pattern was observed in the detection time for abrasions across different wound severities. This is likely because the abrasive stimuli were spatially dispersed, leading to variations in the time required for the model to evaluate them as damage across trials. In contrast, for lacerations, a trend was observed where the time to damage detection decreased as wound size increased. This is thought to occur because larger lacerations exhibit more pronounced structural deviations in the latent space, making them more readily reflected in the damage score. Furthermore, the minimal detection time and variability did not show significant variation across conditions, suggesting the model stably captures internal structural disruption.

In addition, regarding the selection of thresholds (95% and 90%), our quantitative evaluation supports their effectiveness in balancing sensitivity and robustness. As shown in the ROC analysis, the proposed framework achieved an AUC of 0.653, outperforming the simple threshold-based baseline (AUC of 0.511). The high precision (1.0) observed in our experiments indicates that the 95% threshold, combined with the pain-flag gating function, successfully eliminates false positives (FP = 0) even under strong, non-damaging stimuli. While these thresholds are initially derived from statistical distributions of the normal state, the software-driven nature of our model allows for future dynamic optimization based on specific robotic applications and environmental requirements.

Based on the above, the evaluation method proposed in this study demonstrated the potential to more appropriately distinguish between different stimuli and states compared to conventional methods that conflate touch, pain, and damage into a single metric. By explicitly modeling their relationships and temporal dynamics, the method provides a foundation for more interpretable and biologically inspired artificial skin systems. Future work will focus on further refining the representation of damage and extending the framework to more complex and realistic conditions.

### 4.2. Comparison with Neuromorphic and Memristor-Based Systems

The framework proposed in this study offers practical advantages compared to recent neuromorphic systems and memristor-based nociceptive devices. Prior studies on neuromorphic devices have successfully reproduced biological characteristics such as threshold responses, non-adaptation, and sensitization at the device level, providing benefits in terms of high-speed processing and low power consumption. However, these hardware-driven approaches often depend on specific physical device properties, which can limit their scalability and complicate integration into existing robotic systems with diverse sensor configurations.

In contrast, the proposed method is a software-driven approach based on general-purpose capacitive sensors. By employing a convolutional autoencoder (CAE) and ConvLSTM, the model learns the spatio-temporal structure of normal states in an unsupervised manner and defines deviations from this structure as anomalies. This enables the system to tolerate minor variations in sensor characteristics and reduces the need for manual threshold tuning or device-specific calibration. Furthermore, the system can maintain functionality through updates to latent representations and model parameters without requiring hardware redesign, which is advantageous for long-term robotic applications. Another important aspect of this framework is its ability to integrate spatial structural distortions and temporal prediction errors. By incorporating a pain-gating mechanism, the proposed method can distinguish between transient strong stimuli and persistent structural damage, which is difficult for conventional approaches relying solely on signal intensity. This software-based implementation of biologically inspired pain logic provides a flexible and scalable foundation for robotic self-protection systems and may complement existing neuromorphic hardware approaches.

## 5. Limitation

This study has several limitations that should be acknowledged. First, false damage detections were observed under certain conditions even on normal skin, suggesting that temporally concentrated strong stimuli may be misinterpreted as sustained structural changes. In addition, damage scores occasionally approached or exceeded the predefined threshold under normal conditions, indicating that false positives cannot be completely ruled out. Furthermore, intervals were observed in which the touch flag remained active despite the absence of actual contact. This behavior is likely caused by sensor noise, residual charge, or temporal smoothing in the touch map, leading the model to interpret contact as persisting. In Experiment (III), variability was observed in the detection time for abrasions, and no consistent pattern emerged across trials. Moreover, some trials failed to detect damage under certain conditions. These results suggest that the current definition of damage as sustained structural change may not fully capture all types of structural disruptions, particularly those that are spatially dispersed or temporally brief. Another limitation concerns the scale and diversity of the dataset. The training dataset consists of approximately 300 samples primarily based on single-hand contact. Such a limited dataset may restrict the generalization capability of the model. In particular, the effects of diverse contact conditions, including variations in material properties, object shapes, temperature, and environmental noise, have not yet been fully evaluated. Therefore, the present results should be interpreted as a proof-of-concept, and further validation with larger and more diverse datasets is required. In addition, the spatial resolution of the hardware is limited. The current system is composed of a 6×6 grid with 36 electrode intersections, which is sufficient to demonstrate feasibility. However, capturing more complex or fine-grained damage patterns may require higher-resolution sensing. Further investigation is needed to confirm the effectiveness of the proposed framework in higher-density sensor arrays.

Finally, improving robustness to sensor variability and environmental changes remains an important challenge. Future work should focus on enhancing model adaptability and evaluating performance under more realistic and diverse operating conditions.

## 6. Conclusions

This study proposes a novel damage detection method that defines touch, pain, and damage in artificial skin based on normal touch data, then separates and evaluates them as distinct scores. By constructing an anomaly detection model using normal touch maps as a baseline and representing changes in contact states in a latent space, we demonstrate a framework that distinguishes between temporary changes caused by stimuli and structural changes in skin condition.

Experimental results show that the proposed method exhibits distinct responses to different conditions, such as weak touch, strong touch, and damaged skin states. Specifically, it was confirmed that for strong stimuli, pain detection occurs but the state recovers upon stimulus cessation, while for damaged skin, damage detection persists continuously. Quantitative evaluation further validated the system’s performance, achieving an Area Under the Curve (AUC) of 0.653 and a Precision of 1.0 under strong stimuli, significantly outperforming a traditional threshold-based baseline. These results indicate that this method does not rely solely on stimulus intensity for detection but can evaluate changes in skin condition based on temporal persistence and structural features. However, false detection of damage due to sensor irregularities was also observed on normal skin, indicating that stabilizing the damage score in normal conditions remains a future challenge.

Based on the above, this study presents a novel perspective for damage detection by internally representing touch, pain, and damage differently from the touch map of normal artificial skin and evaluating them in an integrated manner. Unlike hardware-dependent neuromorphic systems, our software-driven architecture provides superior scalability and ease of implementation. This approach is expected to be applied as a foundational technology for safe contact control and self-protection mechanisms in robots and wearable devices by adapting to diverse stimulation patterns and detecting degradation during long-term use.

## Figures and Tables

**Table 1:** Confusion metorices and key metric values for the proposed method and the baseline method.

	Confusion Matrix				Metrics			
	TP	FP	TN	FN	Accuracy	Precision	Recall	F1
Proposed Method	117	0	346	220	0.6779	1.0000	0.3472	0.5154
Baseline Method	116	109	237	221	0.5168	0.5156	0.3442	0.4128

**Table 2:** The number of damage flag switches for the proposed method and the baseline method under normal and damaged conditions.

	Situation	
	Normal	Damaged
Proposed method	0	1
Baseline method	9	11

**Table 3:** Confusion metorices and key metric values for the proposed method when changing hyperparameters.

Hyperparameters		Confusion Matrix				Metrics				
λ	α	TP	FP	TN	FN	Accuracy	Precision	Recall	F1	AUC
0.8	0.8	117	0	346	220	0.6779	1.0000	0.3472	0.5154	0.6527
0.2	0.8	188	100	237	152	0.5168	0.6528	0.5529	0.2967	0.4265
2.0	0.8	229	0	338	115	0.8314	1.0000	0.6657	0.7993	0.6221
0.8	0.2	223	0	340	115	0.8304	1.0000	0.6598	0.7950	0.6603
0.8	1.0	0	0	353	339	0.5101	0.0000	0.0000	0.0000	0.5541

## Data Availability

The original contributions presented in this study are included in the article. Further inquiries can be directed to the corresponding author.
